# With Our Editors

**Published:** 1906-12-15

**Authors:** 


### WITH OUR EDITORS.

**POLITICS IN DENTAL SOCIETIES.** A criticism which has long since been worn threadbare is frequently made of there being too much politics in dental societies. A rational study of this question will show to a thinking mind that there always must be politics in the management of any public organization. This is not more true of dental societies than of other societies, that to be prosperous and to be well conducted a society must be under the general management and influence of members of ability in this direction. Without such management and co-operation men will be put into office for the management of affairs who may not be equal to the occasion, either from lack of ability or experience in that direction.

This is especially true of dental societies. When such an organization is mismanaged, which sometimes occurs through placing the wrong man in office, the remedy is easy because by a majority vote the officers are selected or changed.

We have heard such criticism made of the best governed societies of this country. It generally comes from the individual who stands and looks on and is a barrier from his negative aspect, or has come in new, has his toes tramped on and and goes home and nurses his resentment for the rest of his life and therefore takes no active part in the general good of the society.

We are penning these comments at this time especially on account of the severe criticisms that have been made for the last two or three years in regard to the management of the National Dental Association, and while some of these criticisms, we believe, have foundation of fact, the fault is not with the Association nearly so much as it is with our different local and state societies, in that they take little or no interest whatever in this national organization, which should be the most important of all for the entire dental profession of this country.

As is well known, the membership of this Association is composed of delegates from local and state societies. Each society is entitled to one delegate for every five permanent members in its own organization, and it is optional with each delegate whether he becomes a permanent member or simply acts as a delegate and member for the current year. He has only to declare his desire for permanent membership, but despite this liberal method of swelling its ranks the National Dental Association has on its permanent list only about 350 members, instead of which there should be at least ten times that number. In fact, it ought to have 5,000 permanent members that would go to the meetings, bringing with them good material for digestion and taking an active interest in the work. Taking this view of the case, instead of advising members of the profession not to attend the annual meetings, we would advise exactly the opposite, for reasons that must be already apparent to the reader.

Supposing for instance that every society in this country sent its representative men, thus bringing together what has been accomplished for good in all these societes during the year; let us try to imagine what the result would be with four days of literary communion of all the dental profession in this country that amounts to anything, this representing the membership of the different societies.

Tinkering with the by-laws and with the plans of the organization has been indulged in from time to time, the last experiment along this line being to change the plans so that the organization was almost wholly split up into sections, thus patterning after the American Medical Association, without regard to the fact that the latter has a membership of some 20,000, which made the section plan absolutely necessary in its case, owing to the immense amount of work to be covered and the large number of those in attendance at its meetings.

In our opinion, the direction in which to use energy and the reform spirit should be toward obtaining a larger membership, which as we have shown, can be achieved by having the local societies delegate men that will attend the meetings, stand by the Association under all conditions, and not simply use it as a means of obtaining honors, to be followed later by a lapse of membership.

Having been an active member of the National Asssociation for forty years and having missed but one of the annual meetings, and that on account of sickness, and having held the important and laborious office of chairman of the executive committee for over thirty years, we will be given credit for having given this subject some thought. We freely admit, however, having accomplished but little in comparison to the amount of energy and labor expended and are obliged to admit the humiliating fact that the National Dental Association falls far short of what it should be. The fault, however, has not been so much with the management in former years, but may be attributed in large measure to the lack of interest in our national organization in the quarter where such interest should really be most in evidence, namely, the local society.—J. N. Crouse in November *Digest.*

**NARROWING OF THE FOREIGN FIELD FOR AMERICAN DENTISTS.**

For obvious reasons commercial relations with foreign nations must of necessity be far more subject to mutation than is domestic commerce, and this is of course true of that kind of commerce which deals with professional service rather than with the more tangible products of art and industry.

In the early history of dentistry in America those practitioners having highest repute came to these shores from France and England, and found here a profitable field for the exercise of their skill as operators. They also found many eager to become their students. From these beginnings was soon created a corps of dentists of American birth, who had so bettered the instruction of their teachers that the foreign world began to recognize American dentist as the most highly skilled of all practitioners of the dental art, and to seek their services in preference to those of any other nationality.

The demand thus created was soon supplied by the removal to other countries of many American practitioners of high professional ability, some, but not all, having that forcefulness of character and adaptability to new conditions necessary to success in foreign communities.

To this movement the rapid growth of dental colleges gave a great impetus, and during the last fifty years hundreds of young graduates have sought success and fortune in foreign lands.

Of these many have returned disappointed and disillusioned; but large numbers have been reasonably successful while a few have attained to wealth and distinction.

Through a variety of causes the broad field thus opened to American dentists has during the past two decades been gradually narrowing. Chief among these causes is the large number of foreign dental students who, attracted by the fame of American dental schools, have entered as matriculates to learn American methods, and who, after graduation, have returned to become practitioners in their respective countries.

Then too in nearly every European State dental schools have been established, either independent in organization or as departments of universities. While a majority of these are not up to the American standard of instruction, especially in practical operative dentistry, they send out large numbers of graduates sufficiently skilled to meet the present, not too exacting, requirements of the masses of their countrymen.

Inspired by obvious considerations of self-interest both schools and practitioners have of late years sought by restrictive legislation to fortify themselves against the competition of American schools and their graduates. This effort has been so far successful that in a majority of European States the American degree is no longer recognized as an authoritative credential, entitled to full governmental recognition, and its holders are admitted to practice only after compliance with conditions often so onerous as to be prohibitory.

In many South American States also, once freely open to American dentist, formidable obstacles are interposed to the securing of the rights to practice by them or by other foreigners. In Brazil, the largest of the Latin American republics, the requirements, both in time and money, are particularly exacting. Examinations conducted by a medical board in the Spanish language must be passed, the fees and other expenses being very heavy.

Through a letter from Brazil to the *New York Medical Journal* (September 29, 1906), giving an account of the provisions of the Federal law relating to foreign physicians now in force in all the Brazilian States, some idea of the requirements for foreign dentists also may be gained. While these requirements are much less exacting for dentists than for physicians, they differ only in degree. According to the writer of this letter foriegn physicians in Brazil are required to comply with the following conditions:

"*First.*—Present a diploma from some recognized medical college or university, endorsed by the minister or consul of Brazil to the country from which they come, and bearing revenue stamps to the amount of about $40.00.

*Second.*—A letter of identification from some well-known person.

*Third.*—A statement from the judge of the criminal court where they are residing in Brazil that there is nothing in the records of the court against their character.

"These documents having been presented to the secretary of one of the two national medical colleges (there are but two medical colleges in Brazil) in the month of November, the faculty appoints a day in December when an applicant may appear to begin the examinations, which extend over three or four months. The examinations are in four series, the first two being written, oral and practical upon each subject; the others are practical only.

"There are eighteen distinct examinations, on as many different days. There is a different examining board, composed of three members of the faculty, for each subject and a fee of about $35 is paid for each series. There is an additional fee of about the same amount, payable when the diploma is approved. Failure to pass satisfactorily any examination invalidates all. Thus the fees provided by law amount to nearly two hundred dollars, besides which must added the expense of living from three to four months months in Rio de Janeiro or Bahia. It is rarely that a man gets through for less than a thousand dollars, and many spend twice as much, and when one does not pass the first time the expense is almost double. One dentist I knew spent about $3,000 before finally getting his diploma approved.

While through the causes above outlined the field for dental practice in foreign lands is rapidly narrowing, for the American Dentist there is abundant compensation in the fact that the home field is as rapidly broadening. With nearly a million emigrants coming annually to our shores, added to the enormous annual growth through natural increase of population now nearing the one-hundred-million mark, there is in our own country an abundant and ever-widening field for the skilled dental operator. Through the advances in the requirements of dental schools and other contributory causes, the past two years have shown a lessening of the aggregate number of dental graduates of more than fifty per cent., thus to that extent diminishing the number of new competitors. For well qualified men alluring opportunities in foreign lands still occasionally present themselves; but for the vast majority of American practitioners who seek a field for their work life America must long remain the land of wisest choice—Dr. W. F. Litch, in November *Brief*.

